# Conceptual and Methodological Pitfalls in Experimental Studies: An Overview, and the Case of Alzheimer’s Disease

**DOI:** 10.3389/fnmol.2021.684977

**Published:** 2021-06-15

**Authors:** Daniela Puzzo, Fiorenzo Conti

**Affiliations:** ^1^Department Biomedical and Biotechnological Sciences, University of Catania, Catania, Italy; ^2^Oasi Research Institute-IRCCS, Troina, Italy; ^3^Department of Experimental and Clinical Medicine, Section of Neuroscience and Cell Biology, Università Politecnica delle Marche, Ancona, Italy; ^4^Center for Neurobiology of Aging, INRCA IRCCS, Ancona, Italy

**Keywords:** scientific method, cognitive bias, *vera causa*, translational research, Alzheimer’s disease

## Abstract

The main goal of scientific research is to uncover new knowledge to understand reality. In the field of life sciences, the aim of translational research—to transfer results “from bench to bedside”—has to contend with the problem that the knowledge acquired at the “bench” is often not reproducible at the “bedside,” raising the question whether scientific discoveries truly mirror the real world. As a result, researchers constantly struggle to overcome the dichotomy between methodological problems and expectations, as funding agencies and industries demand expandable and quick results whereas patients, who are uninterested in the epistemological dispute, only ask for an effective cure. Despite the numerous attempts made to address reproducibility and reliability issues, some essential pitfalls of scientific investigations are often overlooked. Here, we discuss some limitations of the conventional scientific method and how researcher cognitive bias and conceptual errors have the potential to steer an experimental study away from the search for the *vera causa* of a phenomenon. As an example, we focus on Alzheimer’s disease research and on some problems that may have undermined most of the clinical trials conducted to investigate it.

## Introduction

Translational or “bench to bedside” research aims to transfer knowledge from basic science to clinical practice. As theorized by Claude Bernard, the founder of Experimental Medicine (Conti, 2001, 2002), the process starts with understanding how a physiological system works and tries to uncover the pathophysiology of a disease in order to diagnose, prevent and cure it. The drug discovery process is guided by preclinical studies, where the efficacy and safety of a compound (or device) are tested in animal models before being tried in humans. However, the whole process depends on the validity of the experimental approach itself, since besides the objective risks and intrinsic difficulties of the bench to bedside transition (Seyhan, 2019), methodological and, more frequently, interpretive mistakes may lead to falls in crossing the “valley of death” (Llovera and Liesz, 2016).

Assuming researcher’s *bona fide* and excluding the pressure from big pharma and funding agencies, data manipulation for personal interests, and psychiatric diseases, a number of problems still have the potential to skew research outcomes in any field of science. However, in translational research the challenge and pressure are felt especially keenly, considering the expectations of clinical application and their public health implications. To prevent the failure of the translation process, several studies have examined the main confounding factors that might invalidate an experimental study (see Table 1), focusing on result reproducibility and reliability (e.g., National Academies of Sciences, Engineering, and Medicine (NASEM)., 2019). However, there are few works discussing how to avoid methodological errors due to researcher cognitive biases (Kaptchuk, 2003) and general conceptual errors. Moreover, in life sciences the issue of the scientific method is often dismissed, based on the widespread belief of the existence of only one, undoubtedly correct method (Wagensberg, 2014).

**TABLE 1:** Common errors made in the different phases of an experimental study.

Phases	What to check	Common errors
Before the study	Previous literature	• Poor knowledge of previous studies • Biased interpretation of the existing literature, i.e., exclusive focus on literature supporting our hypothesis, uncritical revision, quality judgment of previous studies exclusively based on journal impact factor
	Choice of the model	• Model not adequate for the purpose of the study • Intrinsic limit of the chosen model
	Experimental design	• Experimental design unclear • Experimental design non-consequential • Experimental design not suitable to achieve the proposed objectives
	Feasibility	• Discrepancy between what you want and what you can do in terms of time, technical, financial and human resources
During the study	Sample size	• Minimum sample size to obtain statistically significant results not calculated • Unclear difference between a pilot study and a study to test a hypothesis • Wrong interpretation of “reduction” in animal research
	Inclusion and exclusion criteria	• Unclear definition of inclusion and exclusion criteria • Overlapping inclusion and exclusion criteria • Selection of inclusion criteria not related to the purpose of the study
	Randomization	• Randomization criteria not established before to start the experiments • Ambiguous randomization criteria • Randomization based on incorrect baseline information or ineligible samples
	Blinding of investigators	• No one blind in the trial • False belief that preclinical studies cannot be blind • Poor standardization when blinding is not possible
	Positive and negative controls	• Experimental plan lacks positive and negative controls, i.e., in preclinical research: to not include in the study untreated/treated healthy animals, untreated models of disease, shame-operated animals, etc.
	Method reliability	• Unreliable methods • Method not adequate to the purpose of the study • Incorrect application of the method • Modification of an existing method without sufficient evidence of its efficacy and reliability
	Environmental conditions	• Different environmental conditions used throughout the experiments • Poor attention toward environmental factors affecting the experiments, i.e., in animal studies: housing, diet, circadian rhythms, temperature, humidity, noise, etc.
	Quality of materials	• Poor quality or expired materials • Cheap but nor certified chemicals or equipment • Low quality samples
	Intrinsic limitations of techniques	• Poor knowledge of the technique, its use and limitation • Confusion between quantitative/qualitative techniques
	Errors in measurements	• Difficulties to detect random or systematic errors, depending on the measurement and the instrument precision and calibration • Undefined conditions that might affect the measurement • Artifacts
	Evaluation of outliers	• To disregard how outliers might influence or even reverse the results • Inappropriate statistical test if outliers are maintained • Unfixed standard methods to remove outliers
	Replication	• Results cannot be replicated in different samples (control for errors, artifacts, environmental factors, conceptual and practical reliability of previous workplan) • Results cannot be replicated in different models (control for model intrinsic differences, adequacy of the method) • Results cannot be replicated in different labs (control for sampling errors, differences in materials, instruments, environmental conditions, procedures, fraud attempts) • Intrinsic variability of responses in living systems, the differences among populations, the low reproducibility of some aspects are not taken into account
	Operators	• Insufficient operator expertise • Inter-operator variability • Operators not blind
	Variables	• Unclear difference between dependent, independent, extraneous and controlled variables • Chosen variables not appropriate to answer the proposed question
After the study	Results interpretation	• Wrong assumptions inferred from statistical analyses • Systematic and random errors ignored • Negative or unexpected results discarded
	Conclusions	• Conclusions do not answer the study question(s) • Conclusions diverge from existing literature without sufficient explanation • Results are not justified and adequately discussed • Differences between prediction and observation not assessed • Biased evaluation of strengths and weaknesses of the study

Here, we briefly discuss some of these methodological errors, hoping that this overview may be useful, especially to young researchers approaching the world of science and research.

## Researcher Cognitive Biases Mirror the Fallibility of the Human Brain

The premise that best explains how cognitive biases may undermine the interpretation of a phenomenon is the fallibility of the human brain. Indeed, we are cognitively predisposed to interpret facts based on a number of fallible systems, whose aim is probably to facilitate the retention of information and to strengthen the force driving our actions. Accordingly we tend to draw conclusions or to find a quick solution to a given problem even though we lack all the information that is required to do so. Although this attitude is clearly advantageous in some life situations, it is a limiting factor when novel research findings confute our beliefs. *Confirmation bias* (Nickerson, 1998), i.e., our tendency to seek and accept information that confirms our prior opinions, leads us to unconsciously select data supporting our views and to ignore opposing evidence. Furthermore, when confronted with ambiguous or opposite evidence, we tend to reinterpret it, to make it consistent with our beliefs and lend more weight to it. The need for confirmation (Peters, 2020) is closely related to pleasure and satisfaction. Even though the degree of confirmation need is strongly related to self-confidence, motivation, and personality structure, the general tendency to prefer positive to negative outcomes (e.g., the Pollyanna Principle, Matlin and Stang, 1979) usually prevails.

*Confirmation bias* is particularly relevant in scientific research, as demonstrated throughout history by scientists’ resistance to discoveries that challenge a current paradigm.

The first strategy that can help combat this tendency is awareness, which is a key prerequisite for any endeavor that needs impartial observation; impartial, maximally objective observation is a central tenet of scientific research. When conceiving an experimental design, neutrality should inform the entire process, from the generation of the hypothesis to the drawing of conclusions, which may either support or reject the hypothesis. A common pitfall is being unaware of snags and problems, which results in the perpetuation of familiar schemes; indeed, even cutting edge technologies cannot rescue an experiment from inadequate underlying reasoning. Assuming *a priori* the goodness of the expected results and rejecting incompatible or negative data are grave methodological mistakes, since a hypothesis is inherently a proposed explanation of reality, not reality itself. This behavior, which has been called *hypothesis myopia* (Nuzzo, 2015), focuses the attention on data supporting the hypothesis through a variety of mental stratagems.

The second strategy that can help us resist our affective loyalty to a notion is to doubt our own stance, avoiding “denial attitude,” which undermines open-mindedness and prevents us from considering different viewpoints. Reasonable doubt is a pillar of scientific research, whose goal is to acquire knowledge by questioning nature through a continuous testing/proof system (Bernard, 1865). Perseverance should not be confused with *belief perseverance*, whose only consequence is to strengthen our own beliefs even in the face of evidence pointing in the opposite direction. We are prey to this phenomenon, which is sometimes called a backfire or boomerang effect (Howard, 2019), when we perceive a threat to our freedom of thought or action. The adverse consequences of such irrational attitude (e.g., the anti-vax movement, COVID-19 denialism) are there for all to see. Rather, facts that clearly contradict our assumptions should carefully be sifted, to allow us to form an unprejudiced view supported by the analysis of the events that have led to the original conclusion.

## Limitations of Conventional Methodology in the Search for the *Vera Causa*

So much has been written about the scientific method that discussing here its rules, interpretation and limitations would involve inappropriate generalizations, besides being beyond the scope of this overview. However, at least for educational purposes, we feel that it may be useful to summarize some of its aspects, bearing in mind that the debate is still open and that novel variables are continuously being introduced by the philosophers of science (Novikov and Novikov, 2013; Grüne-Yanoff, 2014; Stanford Encyclopedia of Philosophy (SEP)., 2015).

In the current scientific context, “conventional methods” have replaced the authentic search for knowledge and most scientists, us included, have become accustomed to employ established methods accepted by the scientific, or in our case, the life science community. As a consequence, we use and teach this scientific method as “the sole” scientific method, advocating its uniqueness and validity and choosing to ignore that other disciplines (e.g., astrophysics, economics) might employ different approaches. In this way we probably disregard the complexity of the epistemological debate. An example of this recent attitude is the “observe-hypothesize-test” system, which most science textbooks present as “the” scientific method (Blachowicz, 2009) without specifying that such step-by-step algorithm is merely a general rule on how to conduct all investigations, as stressed by the philosopher and psychologist John Dewey in *How We Think* (Dewey, 1933). Consequently, we base our work on a rigid proposition that leaves little room for interpretation or flexibility and favors sectorial observation as long as it is verifiable, even outside its “real” context. Is this what the scientific method really prescribes?

As noted above, the observation and organization of empirical facts is at the heart of our knowledge of nature. Despite the variety of explanations provided by philosophers from medieval to modern times, the differences between inductive/deductive or synthesis/analysis reasoning are still applicable. The relevant flow of thought can be summarized as a bottom-up (observation→hypothesis→theory) or a top-down (theory→hypothesis→observation) approach. These apparently opposite methods are not necessarily exclusive, as demonstrated by Galileo Galilei (1564–1642) and subsequently clarified by the four rules of reasoning of Isaac Newton (1642–1726), who, however, overemphasized induction. Indeed, according to the fourth rule (Newton, 1726) “In experimental philosophy, propositions gathered from phenomena by induction should be considered either exactly or very nearly true not withstanding any contrary hypotheses, until yet other phenomena make such propositions either more exact or liable to exception,” paving the way for the modern debate between inductivism and hypothetico-deductivism (Krajewski, 1977). The latter has become the most common approach, especially in life sciences, as also demonstrated by the IMRAD structure of scientific publications: Introduction (the background generating the hypothesis), Methods (how the hypothesis will be demonstrated), Results (data collection), Analysis and Discussion (conclusions). Interestingly, such linear presentation, which we automatically adopt to describe our research work, rarely corresponds to the process that has actually generated our results (e.g., Grmek, 1973; Schickore, 2008), both in terms of the temporal execution of the experiments and of the conception of the experimental plan. Don’t we often reorganize our data to meet the journal’s or the reviewers’ expectations? Thus, manuscripts are written according to the hypothetico-deductive method, even though we may have applied the inductive approach.

However, hypothesis-driven research involves at least two disadvantages. First of all, it prevents *ex novo* exploration of a phenomenon when there are no previous studies of a topic and a hypothesis cannot be clearly stated. The problem is hardly new. The physiologist Claude Bernard (1813–1878) was aware that researchers often encounter matters about which no “fact” is known beforehand. In such cases, an “exploratory experiment” (*expérience pour voir*) is conducted and becomes the starting point for a hypothesis, which is then subjected to experimental verification. Notably, an exploratory experiment led to his celebrated discovery of the effects of curare (Bernard, 1857, 1865). In more recent times, David Hubel and Torsten Wiesel wrote: “Meanwhile we had begun a completely different set of experiments, ones in which specific questions were asked, as opposed to exploration. It is not that we felt that the kind of science that explores, in the manner of Columbus sailing west, or Galileo looking at Jupiter’s moons, or Darwin visiting the Galapagos (often pejoratively referred to as ‘fishing trips’), is in any way inferior to the science we learn about in high school, with its laws, measurements, hypotheses, and so on. Exploration had dominated our work up to then, since we had certainly had no ‘hypotheses’ as we set about to explore the visual cortex. Neither were we in any way “quantitative” in our approach. The term ‘anecdotal,’ a favorite expression of disdain on NIH pink sheets, probably best describes the nature of most of our work, but the deprivation studies were slightly different in that we did ask somewhat more specific questions, without, to be sure, having anything that a modern study section would call a hypothesis” (Hubel and Wiesel, 1998). For the results of their exploratory studies, which today in all likelihood would neither be funded nor pass the review filter, Hubel and Wiesel were awarded the 1981 Nobel Prize for Physiology or Medicine.

Another problem with hypothesis-driven research is that it may prevent questioning the starting hypothesis even if some “facts” clearly contradict it. In his seminal work, *An Introduction to the Study of Experimental Medicine* (1865), Claude Bernard, who was the first researcher to apply the scientific method to medicine, stressed the importance of “facts,” which allow questioning a pre-existing theory if they have been obtained through rigorous experiments. “When we meet a fact which contradicts a prevailing theory, we must accept the fact and abandon the theory, even when the theory is supported by great names and generally accepted.” Only this continuous induction-deduction or facts-theory interaction can guide experimental science. Interestingly, Bernard was already aware that “proof that a given condition always precedes or accompanies a phenomenon does not warrant concluding with certainty that a given condition is the immediate cause of that phenomenon.” This is the *vera causa* principle of Newtonian philosophy (Stanford Encyclopedia of Philosophy (SEP)., 2015; Scholl, 2020), i.e., the requirement for a cause-effect relationship to be proved by direct evidence before it can be accepted. Hypotheses are not to be rejected, but tested by the criterion of counterproof. If disproved, the hypothesis should be discarded or modified; if proved, the experimenter should still doubt. Some authors who consider this approach limiting, especially where theoretical science is concerned, have conceived consequentialist reasoning (see for example Popper’s Falsificationism theory—Popper, 1963). However, the demonstration that a relationship exists and that it is causally competent and responsible for the effect is still a tenet of experimental biology.

Demonstration of the *vera causa* requires the adoption of appropriate methodological standards to obtain reliable experimental data that provide a credible representation of reality and are able to be replicated. The concept of data robustness has been introduced because the fact that a result is replicable does not entail that it is also reliable, and indicates a result that does not vary irrespective of the experimental method used. Hence, a robust conclusion requires a measure of variability through a certain number of independent repetitions conducted under consistent, controlled experimental conditions. “Scientific control” enables the researcher to isolate the effect of the independent variable: minimizing the influence of other variables reduces experimental errors and experimenter bias.

An outstanding example of this approach is found in Bernard’s studies of recurrent sensitivity. Magendie had observed that, in dogs, pinching or cutting the ventral roots induced pain-like responses and that resection of the appropriate dorsal root abolished them, a mechanism he called “recurrent sensitivity.” The topic became popular and was intensely debated (Conti, 2002). Bernard performed numerous experiments on recurrent sensitivity and resolved contradictions by refining the experimental conditions required for its expression, e.g., time interval between surgery and observation, time since the last meal, general conditions of the animal, species, amount of blood loss during surgery, and degree of opening of the vertebral canal (Bernard, 1858).

Nowadays, randomization, blinding and appropriate controls are the fundamentals of the scientific method, although they are not invariably applied. For example, randomization (assignment to a treatment group by a chance process, to minimize differences among groups) and blinding (the experimenter and/or the patient ignore the group to which the patient has been assigned) are mandatory in clinical research, but are not emphasized in basic or translational studies (Karanicolas et al., 2010). Although a double-blind study of cells or animals seems to make little sense, since both are by definition “blind to treatment,” operator blinding during the entire experimental process, from execution to data analysis, would still avoid several biases (see the previous paragraph), maximizing result validity while also preserving that feeling of suspense and curiosity that should drive all researchers.

More complicated is the use and selection of appropriate controls, given their multifaceted nature. As noted above, controls aim to keep variables as constant as possible, to enable isolation of the cause-effect relationship. In an experimental study, this means either using the same experimental conditions (temperature, humidity, oxygenation, chemical solutions, etc.) throughout the replications or introducing control groups that are exposed to the same generic variables with the exception of the independent variable, i.e., the proposed causal factor. A difference in the results obtained in the experimental group compared to controls is highly likely to identify the cause-effect relationship. Negative (not exposed to the experimental treatment) and positive (exposed to a treatment known to exert the effect) controls are also mandatory. Yet, in numerous studies they are either omitted or inappropriate (e.g., lack of comparison between vehicle, i.e., placebo, and drug treatment or between a healthy and a diseased model). For instance, some studies report that a certain treatment rescues a given deficit even if the animal model used to mimic the disease does not present the deficit being investigated. Although model choice is critical in any field of science, it is especially important in preclinical studies using animal models, due to a number of intrinsic variables: (i) the variability of complex systems; (ii) differences between species (e.g., mouse vs. human); (iii) the clear definition of the aspects of the diseases being modeled; (iv) manipulations that result in disease caused by “unreal” causes. Failure to consider these aspects, especially that a model is by definition a representation of reality, not reality itself, may invalidate our experiments or, worse, suggest that the scientific approach has been unsuccessful, whereas it was our interpretation that was to blame.

## The Amyloid-Beta Hypothesis: An Example of Fallacious Interpretation

Cognitive biases and methodological errors affect several fields of biomedical research, but lately a great concern has been rising over the failure of translational research to find a cure for Alzheimer’s disease (AD), the most common form of dementia arising in mid-late life. AD affects the ability to remember, understand and interact with the environment, slowly eroding the patient’s identity and independence in daily life activities. Given that around 50 million people are affected by dementia worldwide and that their number is expected to climb to 74.7 million by 2030 and 131.5 million by 2050 (Giri et al., 2016), AD is a severe social and economic problem, especially in developed countries, where population aging is most advanced. However, despite significant scientific progress, intense basic and preclinical research efforts have failed to deliver applications for clinical practice. According to a growing number of researchers, we have lost our way by testing drugs based on a rationale that is far from the *vera causa*. In particular, the amyloid cascade hypothesis, which has inspired most of the research work conducted to date, is now being set aside after none of the clinical trials aimed at reducing amyloid beta (Aβ) have succeeded in preventing or slowing down the disease. Yet, thousands of preclinical studies have documented a role for it in AD pathophysiology (Sauer, 2014; de la Torre, 2017; Kepp, 2017; Gulisano et al., 2018; Makin, 2018). Where did we go wrong?

Observation is a pillar of scientific research, whether using the inductive or the hypothetico-deductive method. In the case of AD, the amyloid cascade hypothesis appeared to satisfy both the bottom-up (observation→hypothesis) and the top-down (hypothesis→observation) approach, providing a continuum that has reinforced the observation→hypothesis→observation loop. The earliest “observation,” i.e., the identification of senile plaques with/without neurofibrillary tangles by Alois Alzheimer (Hippius and Neundörfer, 2003), was strengthened by post-mortem studies. At least two additional “facts”—the report that senile plaques are formed by Aβ deposits (Glenner and Wong, 1984) and the discovery of rare hereditary forms of early onset Familial Alzheimer’s Disease (FAD), where genetic mutations of amyloid precursor protein and presenilins lead to increased Aβ production (Levy et al., 1990; Hardy et al., 1998)—made Aβ the key factor in AD. These reports gave rise to the amyloid cascade hypothesis (Hardy and Higgins, 1992), which has prompted a variety of studies aimed at confirming the noxious effect of Aβ on synaptic plasticity and memory as well as its increase and deposition in the AD brain. The obvious next step was to remove it from the brain to rescue memory and cure the disease. However, this “Occam’s razor” strategy did not work.

Given the problems hampering translational research, this failure is not really surprising. What is remarkable is that the Aβ notion thrived for decades (Cline et al., 2018) without changes in its rationale (since Aβ increases, it must be removed) and that pharmaceutical companies, funding agencies and health organizations continued to give strong support to anti-Aβ approaches. The neuroscience community is now split into two main camps. Aβ supporters argue that success is a matter of “timing” (i.e., treatment should start earlier) or “personalized therapy” (i.e., treatment should be provided to Aβ-responders). Their stance, which cannot be rejected *a priori*, may also be ascribed to researchers’ “cognitive bias.” The Aβ critics call for aiming at different targets, such as tau protein, whose increase, hyperphosphorylation, and deposition in neurofibrillary tangles is the other hallmark of the disease. But the underlying reasoning is the same: tau is increased in the AD brain→its levels need to be lowered; preclinical studies support the notion→anti-tau strategies must be translated into clinical practice. Altogether, the orchestra is playing the same score even if the second violin has become the first.

Notably, there is a third group of researchers, smaller and probably inadequately supported, who would like to understand where we lost our way, because if we look at the literature with a neutral attitude it is clear that some observations have been emphasized to buttress the amyloid cascade hypothesis whereas some equally important data that contrast with the hypothesis have been underestimated (Table 2). Please see the following reviews for a detailed description of Aβ facts and studies summarized in Table 2; Reitz, 2012; Herrup, 2015; Gulisano et al., 2018.

**TABLE 2:** Amyloid-beta hypothesis facts.

Overestimated facts	Underestimated facts
Senile plaques formed by Aβ deposits are found in AD brains	Senile plaques can be found in cognitively intact individuals
High Aβ concentrations disrupt synaptic plasticity and memory in preclinical models	Low Aβ concentrations improve synaptic plasticity and memory in preclinical models
Aβ triggers tau pathology	Aβ and tau may act independently
FAD is characterized by the same symptoms as AD	Different types of dementia present the symptoms of AD
FAD is caused by genetic mutations that lead to increased Aβ production	Sporadic AD is not due to genetic mutations directly leading to increased Aβ production
Animal models of FAD are used for AD preclinical research	Animal models of FAD do not mimic sporadic AD
Anti-Aβ drugs rescue the cognitive phenotype in FAD animal models	Anti-Aβ drugs do not work on humans with sporadic AD
The amyloid cascade hypothesis might explain AD etiology	AD is a multifactorial disorder characterized, among other factors, by mitochondrial dysfunction, glucose metabolism, ApoEe4 polymorphism, cholinergic dysfunction and vascular problems and influenced by immunity-related and environmental factors.

To discuss here why decades of research have supported (and continue supporting) this hypothesis is outside the scope of our work, but it would be interesting to answer this question: were previous experiments aimed at unveiling the *vera causa*? Because it appears that we have relied on the “inference to the best explanation” by selecting the simplest hypothesis; yet its simplicity does not make it true. The key question that remains unanswered is: why do Aβ and tau increase in the AD brain? The question is quite relevant, since in physiological conditions both proteins play a major role, contributing to neuronal function and structure (Puzzo et al., 2015; Wang and Mandelkow, 2015; Gulisano et al., 2018, 2019), therefore their pharmacological inhibition is potentially unsafe.

## Conclusion

The debate on the scientific method and its inherent limitations is still animated and is expected to continue as knowledge and technology advance. In any case, we should never forget that “What we observe is not nature in itself but nature exposed to our method of questioning” (Heisenberg, 1958). Therefore, the intrinsic limitation of the method, the significance of model and control, and the differences among methodology, methods and techniques need to be pondered each time we design an experimental plan. As researchers, we should continuously strive to balance rigor and creativity, neutrality and sincere curiosity, and the desire to obtain a result and the need to learn the truth, or at least its reflection.

## Author Contributions

DP and FC contributed to conception of the manuscript. DP wrote the first draft of the manuscript. FC wrote sections of the manuscript. Both authors contributed to manuscript revision, read, and approved the submitted version.

## Conflict of Interest

The authors declare that the research was conducted in the absence of any commercial or financial relationships that could be construed as a potential conflict of interest.
